# Potential utility of longitudinal somatic mutation and methylation profiling for predicting molecular residual disease in postoperative non‐small cell lung cancer patients

**DOI:** 10.1002/cam4.4339

**Published:** 2021-10-19

**Authors:** Hang Li, Ze‐Lin Ma, Bin Li, Yun‐jian Pan, Jia‐Qing Xiang, Ya‐Wei Zhang, Yi‐Hua Sun, Ting Hou, Analyn Lizaso, Yan Chen, Xi Li, Hong Hu

**Affiliations:** ^1^ Department of Thoracic Surgery and State Key Laboratory of Genetic Engineering Fudan University Shanghai Cancer Center Shanghai China; ^2^ Institute of Thoracic Oncology Fudan University Shanghai China; ^3^ Department of Oncology Shanghai Medical College Fudan University Shanghai China; ^4^ Burning Rock Biotech Guangzhou China

**Keywords:** DNA methylation, molecular residual disease, prognostic biomarker, recurrence, resected NSCLC

## Abstract

Growing efforts are being invested in investigating various molecular approaches to detect minimal residual disease (MRD) and predict disease recurrence. In our study, we investigated the utility of parallel longitudinal analysis of mutation and DNA methylation profiles for predicting MRD in postoperative non‐small‐cell lung cancer (NSCLC) patients. Tumor tissues and longitudinal blood samples were obtained from 65 patients with resected stage IA‐IIIB NSCLC. Somatic mutation and DNA methylation profiling were performed using ultra‐deep targeted sequencing and targeted bisulfite sequencing, respectively. Dynamic changes in plasma‐based mutation and tumor‐informed methylation profiles, reflected as MRD score, were observed from before surgery (baseline) to postoperative follow‐up, reflecting the decrease in tumor burden of the patients with resected NSCLC. Mutations were detected from plasma samples in 63% of the patients at baseline, which significantly reduced to 23‐25% during post‐operative follow‐ups. MRD score positive rate was 95.7% at baseline, which reduced to 74% at the first and 70% at the second follow‐up. Among the 5 relapsed patients with parallel longitudinal analysis of mutation and methylation profile, elevated MRD score was observed at follow‐up between 0.5‐7 months prior to radiologic recurrence for all 5 patients. Of them, 4 patients also had concomitant increase in allelic fraction of mutations in at least 1 follow‐up time point, but one patient had no mutation detected throughout all follow‐ups. Our results demonstrate that longitudinal profiling of mutation and DNA methylation may have potential for detecting MRD and predicting recurrence in postoperative NSCLC patients.

## Graphical abstract

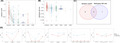

## 1 INTRODUCTION

Lung cancer is an aggressive cancer and the main cause of cancer‐related death worldwide.^1^ For early stage non‐small cell lung cancer (NSCLC) patients, curative surgery has been the standard of care.^2^ Patients with resected stage I NSCLC have better 5‐year survival rate than those with stage IIIA disease (60%–80% vs. 30%).^3^, ^4^ The rate of disease recurrence is about 50% within 10 years in NSCLC patients who underwent surgery.^5^, ^6^ Currently, the eighth edition of the tumor size (T), nodal status (N), and presence of metastasis (M) (TNM) staging system of the International Association for the Study of Lung Cancer (IASLC) has been widely used for predicting prognosis according to the local, regional, and distant extent of the tumor.^4^ However, a few reports have suggested that the current TNM staging system insufficiently reflect prognosis evident by the diverse survival outcomes observed among patients of the same histology and stage.^7^, ^8^ Thus, there is a need to develop accurate prediction model for the recurrence of NSCLC patients. In patients with solid tumors, minimal residual disease (MRD) is conceptually defined as having residual tumor cells after treatments with curative intent, leading to subsequent recurrence and metastasis, which remains to be challenging for both physicians and patients.^9^, ^10^ Hence, detecting postsurgical molecular residual disease is necessary for optimal risk management, which can improve the prognosis of high‐risk patients by tailoring adjuvant therapy and surveillance imaging and minimize unnecessary treatment in patients with low risk of recurrence.^11^

Numerous studies have demonstrated that circulating tumor DNA (ctDNA) is a promising biomarker for molecular profiling, treatment monitoring,^12^, ^13^, ^14^ and MRD detection in lung cancer,^14^, ^15^, ^16^, ^17^, ^18^ colon cancer,^19^ and breast cancer.^20^ Somatic mutation profiling from ctDNA has been extensively explored. However, due to the limited amount of ctDNA present in early stage patients, a significant percentage of patients has no mutation detected from their blood samples.^17^, ^18^, ^21^, ^22^ Collectively, more sensitive and specific method should be explored for predicting the recurrence of postoperative NSCLC patients.

DNA methylation, an epigenetic modification in the context of CpG dinucleotide in mammalian genome, is important in gene regulation^23^ and plays a critical role in cancer initiation, progression, and metastasis.^24^, ^25^ Compared with somatic mutation, some studies have demonstrated that DNA methylation is a more promising biomarker for cancer screening, early detection, and recurrence monitoring due to its early occurrence, abundant signal, and stability.^26^, ^27^, ^28^, ^29^ Field cancerization refers to a field of normal‐like tissue preconditioned by undefined processes, predisposing it toward cancer development.^30^ Genetic and epigenetic alterations have been implicated as the molecular mechanisms of field cancerization and underlie the development and progression of various cancers including lung cancers.^31^, ^32^ A recent study has demonstrated the prognostic value of an individualized methylation‐based analysis of field cancerization by comparing the methylation signatures of tumor tissues and paired adjacent normal tissues of patients with resected stage IA lung adenocarcinoma^33^; however, the analysis of longitudinal blood samples and its utility in postoperative disease monitoring has not been assessed. In our study, we used an individualized methylome‐based predictive model to monitor the risk of recurrence of postoperative NSCLC patients. We further compared the utility of combining the analysis of cell‐free DNA (cfDNA) methylation and ctDNA somatic mutation profiles in detecting MRD for predicting the recurrence risk of postoperative NSCLC patients.

## 2 MATERIALS AND METHODS

### 2.1 Patients

From December 2017 to July 2019, 65 patients diagnosed with resectable NSCLC (IA–IIIB) with various histological subtypes at Fudan University Shanghai Cancer Center were prospectively enrolled in this study. Contrast chest computed tomography (CT), brain magnetic resonance imaging, bone emission CT, and abdominal ultrasound were performed during follow‐ups to detect local recurrence or distant metastases. Tumor assessment was assessed according to Response Evaluation Criteria in Solid Tumors (RECIST) version 1.1.^34^ Written informed consent was obtained from all patients. This study was approved by the ethics committee at Fudan University Shanghai Cancer Center (NO. 1710177‐19).

### 2.2 Sample collection and preparation

Paired tumor tissues were surgically collected. Longitudinal blood samples before surgery (baseline) and at multiple evaluation time points were also obtained. Blood samples (~10 mL) were collected in Streck cell‐free DNA blood collection tubes (Streck). Plasma were obtained from blood samples by centrifugation at 2,000× *g* for 10 minutes. Between 1 and 5 mL of plasma were used for cfDNA extraction. CfDNA and tumor tissue DNA were extracted using appropriate Qiagen DNA extraction kits for tissue and circulating nucleic acid (Qiagen) following the manufacturer's instructions as previously described.^35^

### 2.3 Targeted somatic mutation and bisulfite sequencing

Plasma‐based and tissue‐based somatic mutation profiling were performed using targeted sequencing coupled with unique molecular identifier (UMI)^35^ and capture‐based targeted sequencing,^36^ respectively, using a commercial panel consisting of 168 lung cancer‐related genes (Lung Plasma, Burning Rock Biotech) as described previously. Sequencing of the indexed samples was performed using a NovaSeq 6000 instrument (Illumina,) with 2 × 150 base pair cycles at target sequencing depths of 10,000× for plasma samples and 1,000× for tissue samples. Paired lymphocyte DNA were also sequenced to remove mutations related to clonal hematopoiesis. DNA methylation profiling was performed on bisulfite‐converted DNA prepared using a targeted panel covering 80,672 CpG sites and sequenced using a NovaSeq 6000 instrument (Illumina) as described previously.^33^, ^37^, ^38^ A tumor‐informed MRD prediction model, as described previously, was employed to calculate the corresponding methylation signal intensity of the plasma sample of the patient at baseline and other follow‐up time points based on methylation signals obtained from the patient's resected tumor tissue samples, which is reflected as MRD score.^33^ Sample processing, sequencing, and analysis were performed at Burning Rock Biotech, a clinical laboratory accredited by the College of American Pathologists and certified by the Clinical Laboratory Improvement Amendments. Sequence data analysis^35^ and DNA methylation analysis^33^, ^38^ were performed as described previously.

### 2.4 MRD score calculation

A tumor‐informed methylation‐based MRD prediction model, reflecting the methylation signal intensity of the matched plasma samples, was constructed to predict the recurrence risk of resected NSCLC patients as described previously.^33^ Patients with tumor content of less than 30% were excluded from the MRD score calculation.

Briefly, the cancer‐specific methylation blocks (MBs) were selected by comparing the methylation profiles of tumor and normal plasma samples from healthy donors. MBs with significant difference (*p *< 0.05) were chosen.

Where the unknown parameter *α_i_* denotes the ctDNA fraction in cfDNA, which is defined as MD ratio. Then MD ratio of each patient was estimated by maximum likelihood estimation (MLE), reflecting the proportion of methylation signature in the plasma sample shared by its corresponding tumor tissue. The variance of estimator $\hat{\alpha_{i}}$ was calculated using the Fisher information matrix. MRD score was defined as the Wald statistic under the null hypothesis: $\alpha_{i}=0$, denoting the density of malignant signatures in the plasma sample of each patient.^33^

### 2.5 Statistical analysis

Statistical analysis was conducted using R version 3.3.3 software. The Fisher's exact test or paired two‐tailed Student's *t* tests were applied for comparing difference between groups, as appropriate. Wilcoxon test was used to investigate the clinical relevance of baseline plasma maximum allelic fraction (maxAF) and MRD score and the correlation between baseline plasma maxAF and MRD score. *P* < 0.05 was considered statistically significant.

## 3 RESULTS

### 3.1 Characteristics of patients

**FIGURE 1:**
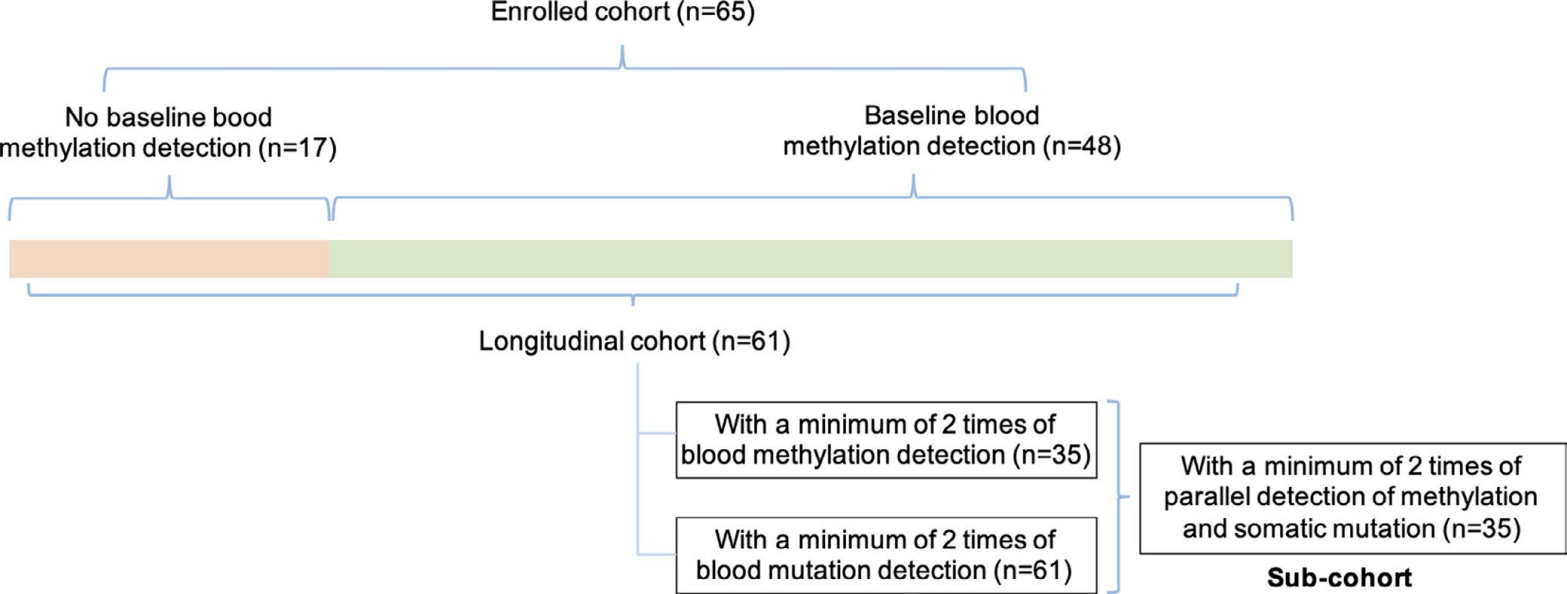
Overview of the cohort. We enrolled 65 patients (enrolled cohort) which all had baseline somatic profiling from both tumor tissue and plasma samples. Among them, 48 patients had methylation profiling from blood at baseline, respectively. Depending on the amount of cfDNA obtained from blood samples at follow‐up visits, patients with sufficient cfDNA were subjected to both somatic mutation and DNA methylation profiling. Patients with limited amount of cfDNA were only subjected to somatic mutation profiling. Blood‐based methylation and somatic mutation profiling were performed on 35 and 61 patients, respectively, who were included in the longitudinal cohort. 35 patients with a minimum of two follow‐ups had somatic mutation and DNA methylation profiling performed were included in the sub‐cohort.

This study enrolled 65 patients with resectable NSCLC having a median age of 60, ranged from 36 to 74 years. Lung adenocarcinoma (LUAD, *n *= 49, 75.4%) and lung squamous cell carcinoma (LUSC, *n *= 11, 16.9%) constituted the majority of enrolled patients. The remaining patients (*n *= 5, 7.7%) were classified as other NSCLC including adenosquamous carcinoma (*n *= 3), large cell carcinoma (*n *= 1), and lymphoepithelioma‐like carcinoma (*n *= 1). Somatic mutation profiling was performed on all baseline tissue (*n* = 65) and the paired blood samples (*n *= 65). Depending on the amount of cfDNA obtained from blood samples, patients with sufficient cfDNA were subjected to both somatic mutation and cfDNA methylation profiling. Patients with limited amount of cfDNA were only subjected to somatic mutation profiling. Of the 65 patients, 65 and 48 patients had DNA methylation profiling performed for tissue and plasma samples, respectively, at baseline. Patients with a minimum of two follow‐up visits were included in the survival analysis. Collectively, 35 patients and 61 patients had a minimum of two cfDNA‐based DNA methylation and somatic mutation profiling performed during follow‐up visits, respectively. The median follow‐up time was 132 days (ranged from 10 to 676 days). The patient demographic and study design are summarized in Table 1 and Figure 1, respectively.

**TABLE 1:** Characteristics of patients

Characteristics		No. (%)
Age (years‐old)	mean: 38 (median: 60, 36–74)	
Sex	Female	26 (40.0)
	Male	39 (60.0)
Pathologic type	LUAD	49 (75.4)
	LUSC	11 (16.9)
	Others	5 (7.7)
Stage	Ia	8 (12.3)
	Ib	4 (6.2)
	IIa	5 (7.7)
	IIb	14 (21.5)
	IIIa	29 (44.6)
	IIIb	5 (7.7)
Smoking history	YES	33 (50.8)
	NO	32 (49.2)
Alcohol history	YES	21 (32.3)
	NO	43 (66.2)
	Unknown	1 (1.5)
Differentiation	High–medium (1)	1 (1.5)
	Medium (2)	11 (16.9)
	Medium–low (3)	26 (40.0)
	Low (4)	27 (41.5)

### 3.2 Landscape of baseline mutations in tissue and blood samples

**FIGURE 2:**
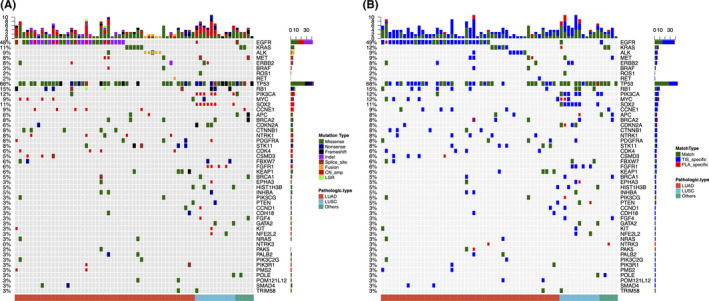
OncoPrints of somatic mutations identified from lung tumor tissue and plasma samples at baseline of the enrolled cohort. A, Somatic mutation profile of lung tumor tissue samples from each patient. The colors denote different types of mutations. B, Somatic mutation profiles of plasma sample compared with the paired lung tumor tissue samples from each patient. Using mutations detected from lung tumor tissue as a reference, different colors denote whether the mutation detected from plasma sample is matched with the lung tumor tissue (Match), detected only from the lung tissue sample and was not detected in the plasma sample (Tis‐only), or detected only from the plasma sample (Pla‐only). Top bars denote the total mutation count of each patient; component bar graphs on the right side denote the distribution of mutation type (A) or for each gene. Bottom bars denote histology types; lung adenocarcinoma (LUAD), lung squamous cell carcinoma (LUSC), and others

**FIGURE 3:**
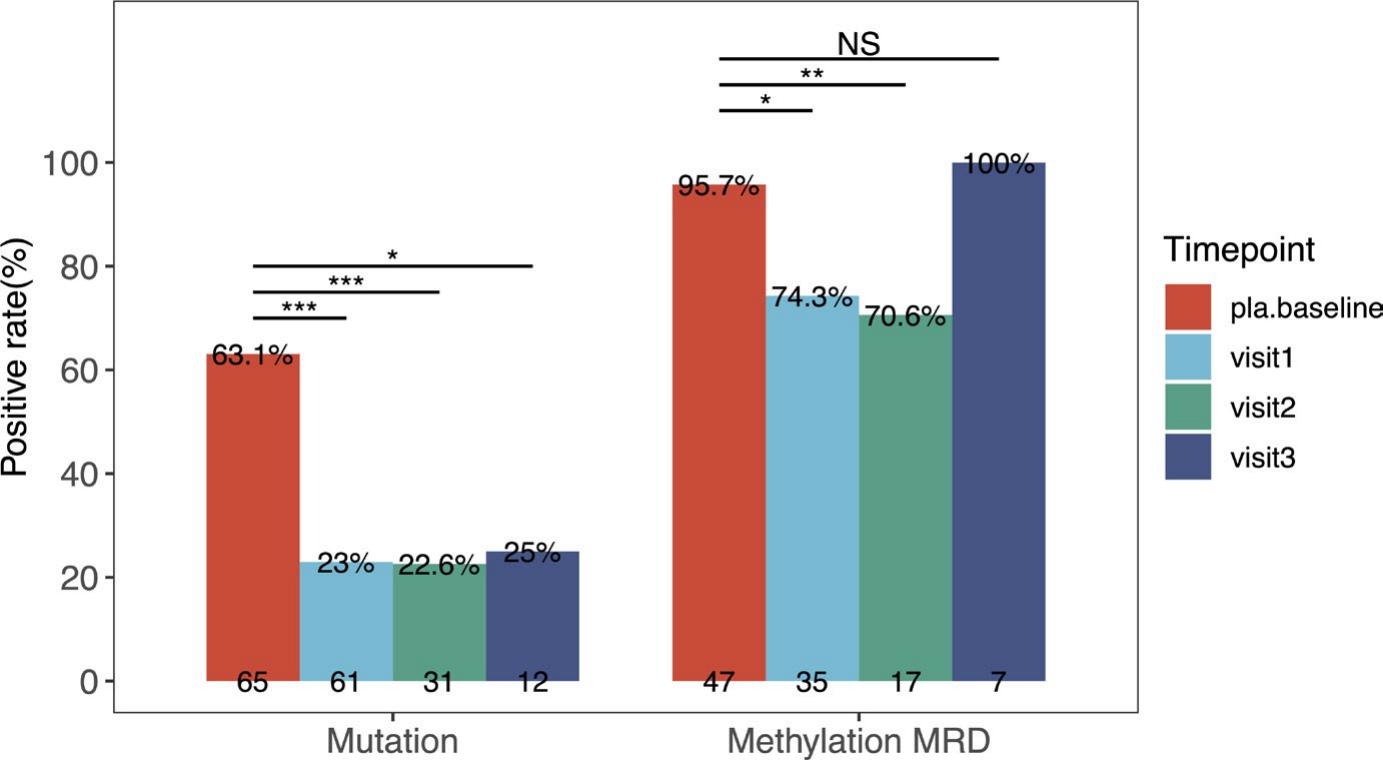
Somatic mutation and methylation MRD of resected NSCLC patients throughout the follow‐up. Positive detection rate of somatic mutation and methylation MRD score. Methylation MRD positive is defined as wald >1.96. Of note, patients with tumor cell fraction less than 30% were excluded from the assessment of MRD score. Comparisons were analyzed by the Fisher's exact test or Wilcoxon test as appropriate. Analysis of variance was applied. *p < 0.05; **p < 0.01; ***p < 0.001

We performed capture‐based targeted sequencing on baseline tumor tissues and the paired plasma samples for detecting genomic aberrations. The baseline mutation landscape is shown in Figure 2. All patients had somatic mutations detected from their tissue samples, while only 63.1% of patients had mutations detected from their paired blood samples, resulting in a concordance rate of 63.1% (Figure 3). From the tissue samples of LUAD patients, the epidermal growth factor receptor (*EGFR*) gene was detected with the most mutations (*n* = 30, 61.2%), followed by Kirsten rat sarcoma virus proto‐oncogene (*KRAS*) (*n* = 5, 10.2%), anaplastic lymphoma kinase (*ALK*) rearrangement (*n *= 5, 10.2%), and catenin B1 (*CTNNB1*) (*n *= 5, 10.2%). While in the LUSC patients, the most common alterations occurred in the tumor protein 53 (*TP53*) (90.9%), followed by gene amplifications of the sex determining region Y box transcription factor 2 (*SOX2*) (*n *= 7, 63.6%), phosphatidylinositol‐4,5‐bisphosphate 3‐kinase, catalytic subunit alpha (*PIK3CA*) (*n *= 6, 54.5%), and fibroblast growth factor receptor 1 (*FGFR1*) (*n *= 4, 36.4%). Approximately 40% of patients had no mutation detected from their baseline plasma samples, indicating the limited sensitivity of mutation detection in early stage patients and suggesting the need for the development of alternative methods.

### 3.3 The landscape of cfDNA methylation profile at baseline

DNA methylation profiling was performed on 48 paired baseline blood and tumor tissue samples. MD scores were calculated to reflect the methylation profile as well as the presence of MRD in plasma samples. Heat map of DNA methylation status of tumor and baseline plasma samples is shown in Figure S1. Next, we investigated the correlation between clinical parameters and maximum allelic fraction (maxAF) or methylation MRD score obtained from baseline plasma samples. MaxAF is defined as the highest fraction of mutant allele detected in a particular plasma sample. Analysis revealed that LUSC patients had higher maxAF (*p *= 0.004) and MD score (*p *= 0.002) compared with LUAD patients (Figure S2A). Higher maxAF and MD score were associated with more advanced tumor (T) stage (Figure S2B) and poor differentiation (Figure S2C). Furthermore, mutation detection rate was positively correlated with pathological stage (*p* = 0.042), while MRD score was not associated with pathological stage, with similarly high detection rate among stage I to stage III patients (90% vs. 100% vs. 95%; Figure S2D). These data suggest that the sensitivity of tumor‐informed methylation analysis is higher than mutation detection and can allow detection even in blood samples from stage I patients.

### 3.4 Dynamic changes in ctDNA mutation and DNA methylation profiles from baseline to various postoperative follow‐up time points

We first investigated the dynamic changes in somatic mutation and DNA methylation profiles of blood samples from our cohort before surgery and at various postoperative follow‐up time points to understand their feasibility in reflecting MRD. As compared to baseline, ctDNA mutations were significantly reduced at first follow‐up (23% (14/61); *p* < 0.001), at second follow‐up (23% (7/31); *p* < 0.001), and at third follow‐up (25% (3/12); *p* = 0.024; Figure 3). Twenty‐four patients who were mutation positive at baseline had ctDNA clearance at first follow‐up after surgery. CtDNA clearance was defined as the lack of detectable mutation from the panel used. Twenty‐four patients had no somatic mutation detected from baseline and other follow‐up time points. Overall, 10 patients had elevated maxAF during the follow‐ups, with 6 patients confirmed to have disease recurrence and 4 patients remained recurrence‐free. As of May 2020, eight patients (12.3%, 8/65) had been confirmed to have disease relapse by radiological imaging. Of them, two relapsed patients were not detected with somatic mutations throughout the follow‐up.

Methylation MRD positive is defined as wald >1.96. As compared to baseline, detection rate of methylation MRD was significantly reduced at first follow‐up (74.3% (26/35); *p* = 0.007) and second follow‐up (70.6% (12/17); *p* = 0.011; Figure 3). However, elevations in detection rate of MRD scores were observed at third follow‐up, with detection rate of 100% (7/7). Among the eight patients with recurrence, five patients had at least two time samples analyzed for DNA methylation. All these five patients were detected with elevated MRD scores at second follow‐up (*n* = 5).

Analysis revealed a positive correlation between maxAF and methylation MD score of each sample (correlation coefficient of 0.89; *p* < 0.001; Figure S3), suggesting the feasibility of using both maxAF and MD score in reflecting MRD.

Taken together, these data indicate the potential of cfDNA‐based analysis of somatic mutation and methylation in reflecting MRD.

### 3.5 Prognostic value of parallel analysis of ctDNA‐based mutation and DNA methylation profiles

We further investigated the potential prognostic value of the parallel analysis of both somatic mutation and DNA methylation profiles for predicting MRD. To compare the prognostic value of both maxAF and MD score, we analyzed a sub‐cohort consisting of 35 patients with matched somatic mutation and DNA methylation profiles at a minimum of two follow‐up time points.

**FIGURE 4:**
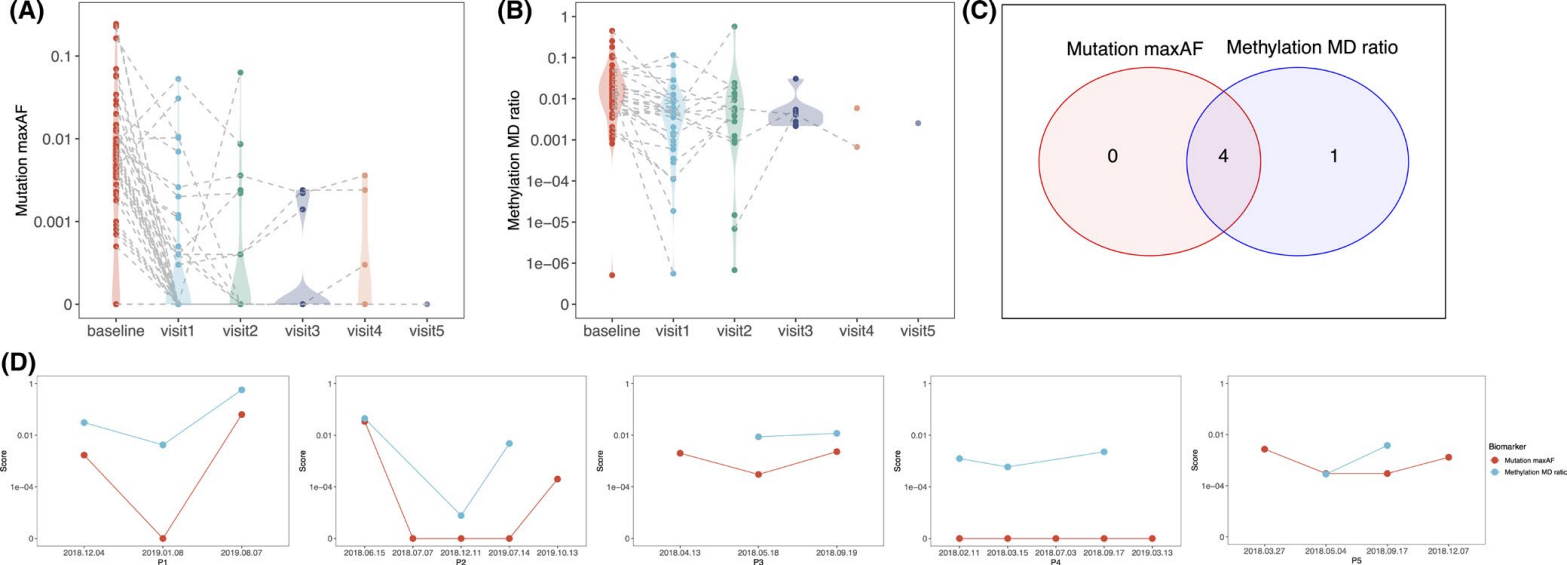
Longitudinal plasma maxAF and methylation MRD model for postoperative disease monitoring of patients. To compare the predictive value of maxAF and MRD score model, we analyzed a subset of patients with a minimum of two follow‐ups had somatic mutation and DNA methylation profiling performed. Longitudinal plasma maxAF (A) and methylation MD score (B) of resected NSCLC patients. C, In this sub‐cohort, disease relapse was radiologically confirmed in five patients ranged from 5 to 14 months post‐surgery. D, Serial monitoring of maxAF and methylation in the plasma of the five patients whose relapse was confirmed radiologically. Yellow line denotes the time of radiological recurrence. Recurrence is predicted by the elevation of either maxAF or MD score.

As compared to baseline, 65% (13/20) of patients had ctDNA clearance at the first follow‐up post‐surgery (Figure 4A). At second follow‐up, two and seven patients were detected with an increase and a decrease in maxAF, respectively, compared with the first follow‐up. MD scores were significantly reduced at first follow‐up (82.1% (23/28); *p* = 0.003) and second follow‐up (63.6% (7/11); *p* = 0.101). At second follow‐up, six and five patients had an increase and a decrease in MD score, respectively, compared with the first follow‐up (Figure 4B).

In this sub‐cohort, five patients had radiologically confirmed disease recurrence ranging from 5 to 14 months post‐surgery (Figure 4C). All these five patients had elevated MD scores at any follow‐up time point, occurring between 0.5 and 7 months prior to radiologic recurrence. Among these five patients, one had no somatic mutation detected in any follow‐up time points. The remaining four patients had elevated maxAF prior to radiologic recurrence, instead of increased number of mutations, as compared to their sample from previous follow‐up time point. Of them, two patients had elevated MD score prior to elevation of maxAF, while the other two patients had both elevated maxAF and MD score at the same time points (Figure 4D).

## 4 DISCUSSION

In this study, we comprehensively investigated the prognostic value of cfDNA methylation and somatic mutation profiling in postoperative NSCLC patients. This study demonstrated that the combined longitudinal analysis of cfDNA methylation and somatic mutation could reflect MRD at the molecular level and identify the patients with high risk of recurrence. It may serve as a robust method for disease monitoring and predicting the recurrence of NSCLC patients.

Disease recurrence is conventionally diagnosed by imaging techniques, which are incapable of detecting MRD until a sufficient tumor bulk can be observed.^19^ Recent studies have shown that somatic mutation in ctDNA has a potential for predicting the recurrence.^16^, ^19^ Due to the limited amount of ctDNA present in early stage patients, a substantial number of patients have no mutation detected from their blood samples even before surgery. Detection limit for regular NGS assays is insufficient for analyzing such low‐frequency variants in cfDNA; the use of UMI‐based capture probes has been demonstrated to enable sequencing depths to reach up to 50,000× and improve the accurate detection of mutations with ultralow frequency of up to 0.05% from blood samples of patients with early stage lung cancer.^35^ UMI‐tagging of ctDNA fragments from early stage patients improves the accuracy of detecting ultralow frequency mutations by allowing the distinction between the original DNA template and contaminants, which minimizes false detection of somatic mutations.^35^ In our study, 63.1% of patients had somatic mutation detected at baseline from plasma as compared with 100% from paired tumor tissues. Despite the use of UMI‐based sequencing, the lower detection of somatic mutations from blood samples of early stage patients was due to the smaller tumor bulk and the lower concentration of ctDNA present in their circulation, particularly from patients with stage IA disease and LUAD histology as reported by Yang et al.^35^ Our cohort was also comprised of 75% LUAD, which had been shown as a histology with lower plasma‐based mutation detection rate and lower tissue to plasma mutation concordance as compared with LUSC.^39^ Among the eight relapsed patients confirmed by radiologic imaging in the enrolled cohort, one patient had no somatic mutation detected from his plasma sample throughout the follow‐up period, and another patient had ctDNA clearance. However, our data demonstrates that the sensitivity of plasma‐based somatic mutation profiling alone is very limited in predicting disease recurrence, particularly for patients who have lower ctDNA levels, which suggests the need to explore alternative molecular assays that could further improve the sensitivity in detecting MRD and identify patients who are at high risk of disease recurrence.

Since DNA methylation occurs very early during the process of carcinogenesis, it is now considered as a promising tool for cancer screening, early detection, and recurrence prediction.^26^, ^27^, ^28^, ^29^ Dynamic changes in DNA methylation were demonstrated to be positively correlated with tumor burden and clinical response to therapy, and can also be detected prior to radiologic confirmation of disease recurrence or progression, implying that methylation in ctDNA could be a viable biomarker for detecting MRD and monitor therapeutic response.^33^, ^37^, ^40^, ^41^, ^42^, ^43^, ^44^, ^45^ Numerous studies have demonstrated that the methylation status of selected number of genes from blood and other biological samples had prognostic value in stage I NSCLC.^15^, ^16^, ^17^, ^18^, ^26^, ^27^, ^46^ However, selection bias may contribute to inter‐patient and intra‐patient heterogeneity, which could skew the results from using a tumor‐naive approach in the analysis of methylation status. In addition, those studies were also limited by the use of a small panel of genes. Recently, an individualized tumor‐informed MRD scoring system was constructed which compared the DNA methylation signatures of the paired tumor and adjacent normal tissue from the same individual in order to identify tumor‐specific epigenetic changes in plasma samples.^33^ A comprehensive DNA methylation sequencing panel interrogating 80,672 CpG sites coupled with the tumor‐informed MRD analysis was shown to accurately predict the recurrence risk from tissue samples of a cohort with resected stage IA lung adenocarcinoma.^33^ The tumor‐informed approach, by taking into account the methylation signal intensities from the patient's own paired tumor and normal tissues, can greatly minimize the inter‐patient heterogeneity in methylation signal intensity and facilitate accurate identification of tumor‐specific methylation signals.^33^ In our study, we have extended the potential of this approach in the longitudinal analysis of blood samples and investigated its feasibility in monitoring the disease recurrence of patients with resected stage IA–IIIB NSCLC. In a subset of patients who had data for both somatic mutation and DNA methylation profiling at a minimum of two follow‐up time points, all the five relapsed patients had elevated MRD score as early as 7 months prior to their radiologic recurrence. This finding suggests that DNA methylation profiling alone is sensitive in detecting early disease recurrence. The detection of somatic mutations at pre‐surgical or postoperative time points had been consistently associated with poor prognosis.^14^, ^26^, ^27^, ^28^, ^29^ Our findings also suggest that the combination of somatic mutation and DNA methylation profiling improves the specificity and sensitivity of the longitudinal, individualized blood‐based risk prediction in our cohort. TNMB, which integrates the TNM staging system and the tumor biology (B), has been proposed as a novel prognostic classifier and may provide a better estimate of the prognosis of patients with resected NSCLC than the conventional TNM staging system.^47^, ^48^ We speculate that longitudinal blood‐based tumor‐informed genomic and epigenetic profiling coupled with other serum biomarkers, clinical symptoms, and history could contribute to a more accurate risk stratification of patients with resected NSCLC. Our study contributes to the growing evidence that DNA methylation profiling may be a valuable real‐time biomarker for predicting MRD and can be used together with the clinical symptoms, traditional imaging modality, and somatic profiling to monitor disease recurrence of postoperative NSCLC patients.

Our study has limitations due to the inclusion of a small cohort and short follow‐up time. Some patients who had elevated MRD scores might experience relapse after sufficient follow‐up time. Studies with larger cohorts, consistent sampling time points at follow‐up, and longer follow‐up time are required to validate the prognostic value of the MRD score and improve the robustness of the prediction model. Our study was also limited to the investigation of both somatic mutation and DNA methylation profiles and did not include the analysis of clinical status or other biomarkers that could further improve the sensitivity of the risk prediction model. Nonetheless, this study reveals that parallel longitudinal assessment of DNA methylation and somatic mutation has potential for predicting the recurrence in patients with resected stage IA–IIIB NSCLC. The parallel assessment of DNA methylation and somatic mutation can potentially improve the sensitivity in identifying patients with higher risk of recurrence as compared with imaging modalities or somatic mutation profiling alone. Our study contributes an incremental step in developing strategies for risk stratification of NSCLC patients after surgery.

## CONFLICT OF INTEREST

All authors have declared that no competing interest exists.

## ETHICAL STATEMENT

The authors are accountable for all aspects of the work in ensuring that questions related to the accuracy or integrity of any part of the work are appropriately investigated and resolved. This study was approved by the ethics committee at Fudan University Shanghai Cancer Center (NO. 1710177–19).

## Supporting information

Fig S1‐S3

## Data Availability

The datasets generated during and analyzed during the current study are available from the corresponding author upon reasonable request.
