# Supplementary material for: Potential utility of longitudinal somatic mutation and methylation profiling for predicting molecular residual disease in postoperative non‐small cell lung cancer patients
Source: Cancer Med. 2021 Oct 19;10(23):8377–86. doi: 10.1002/cam4.4339 (PMC8633238; doi:10.1002/cam4.4339)
Supplement: Supplementary file 1 — Fig S1‐S3 [file CAM4-10-8377-s001.docx]

**Table of contents**

**Supplementary Figure 1.** Heat map of DNA methylation status of tumor and baseline plasma samples.

**Supplementary Figure 2.** Clinical relevance of baseline plasma mutation and methylation

**Supplementary Figure 3.** Correlation between baseline maxAF and MRD score

**
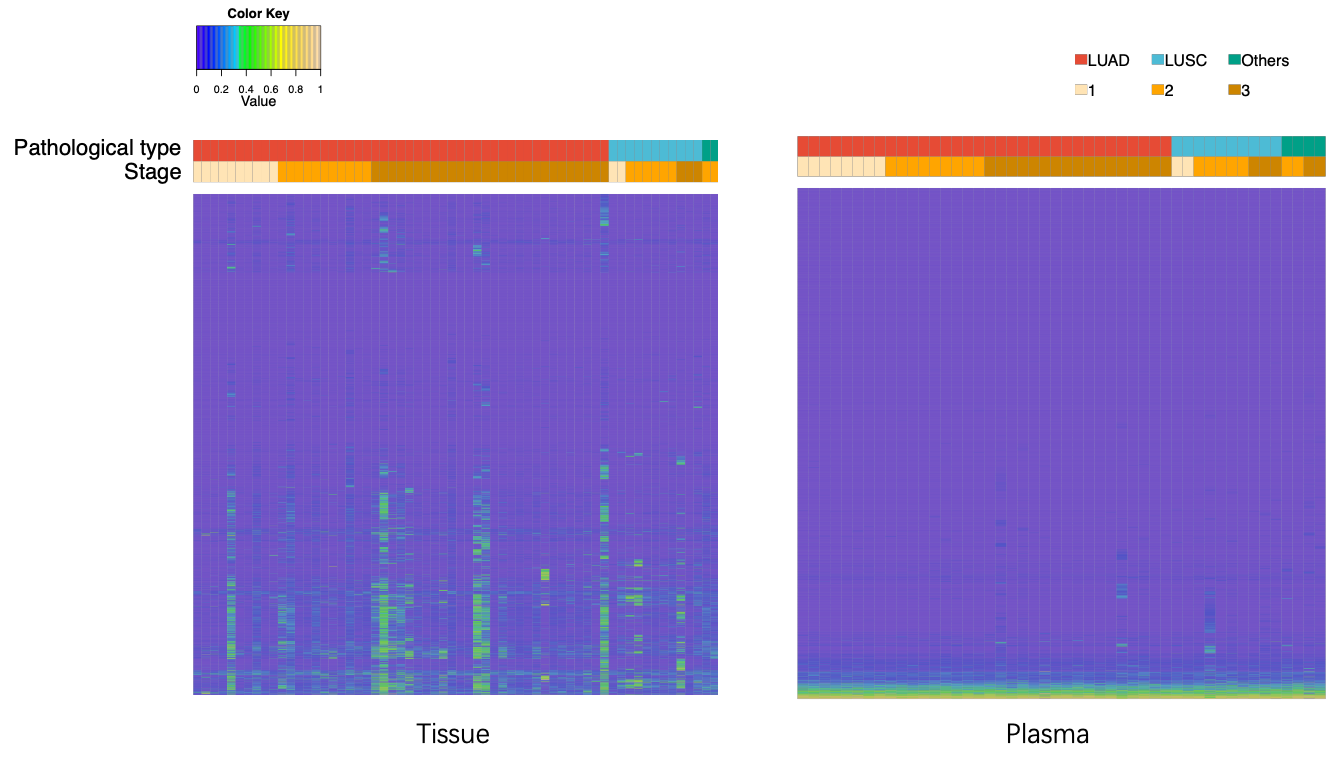
**

**Supplementary Figure 1.** Heat map of DNA methylation status of tumor and baseline plasma samples. Methylation blocks (MBs) were defined as the genomic region consisting of the neighboring CpG sites that have correlated methylation level. A total of 8,312 MBs were generated from 80,672 CpG sites. Heat map score mapping of 65 tumor tissue and 48 baseline plasma DNA samples. Each row represents one methylated block and each column represents one sample. Colors indicate the DNA methylation level for each MB per sample, with the higher value represented as yellow. The annotations above indicate the pathological subtype and stage of each patient.


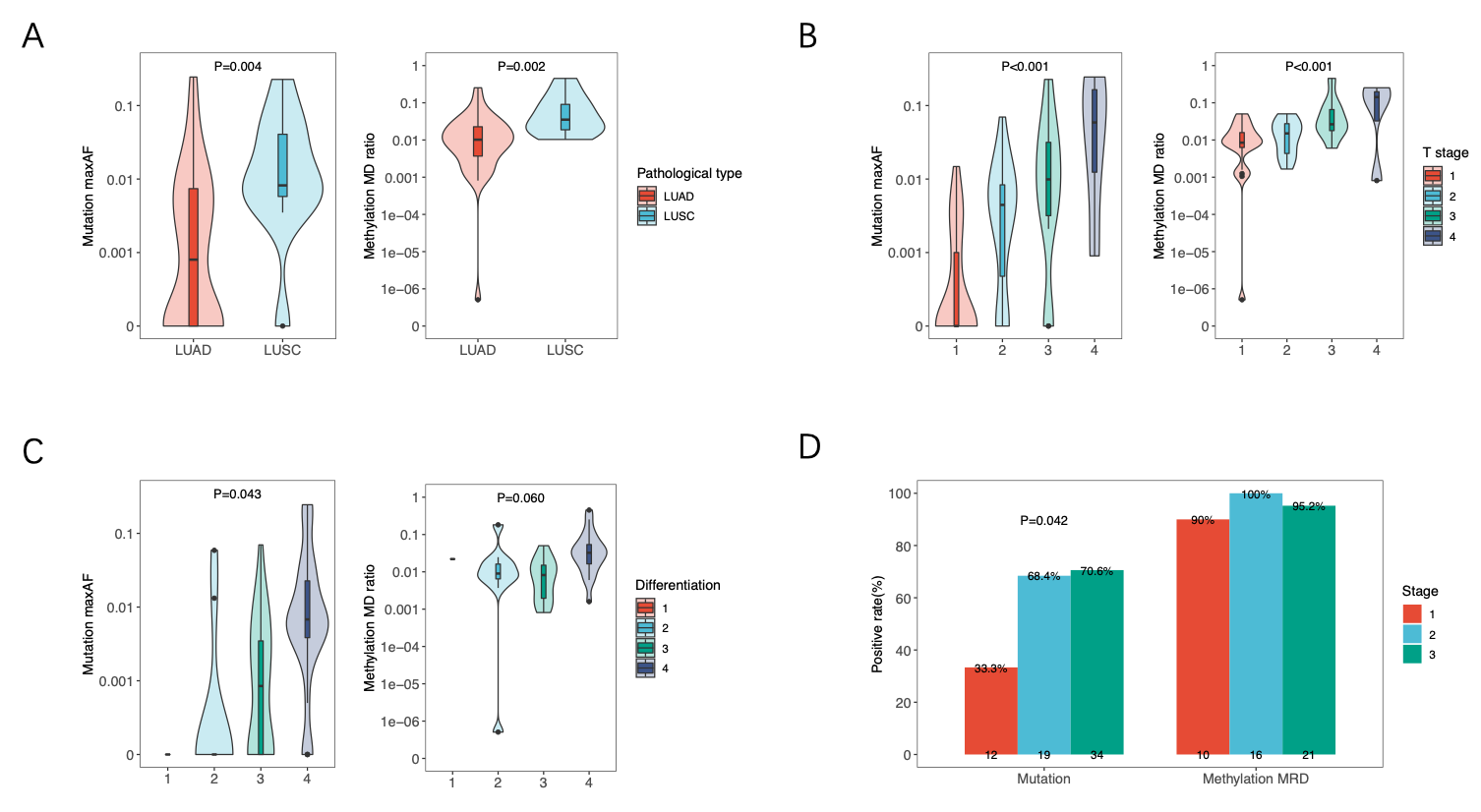


**Supplementary Figure 2.** Clinical relevance of baseline plasma mutation and methylation profile. Clinical characteristics (Pathological type (A), T stage (B), differentiation degree (C), pathological stage (D)) associated with maxAF and methylation. Differentiation degree 1，2，3，4 represents high-median, median, median-low, low differentiation, respectively. Patient with tumor cell fraction of tumor tissue less than 30% was excluded from methylation MRD model. Pearson or wilcox correlation test was applied for continuous variables or binary variables, respectively. Plots of both variables over the dichotomized clinical features are shown.

**Supplementary Figure 3.** Correlation between baseline maxAF and MD score
